# Diagnostic Factors Associated with Sarcoidosis in Patients Referred for EBUS-TBNA Due to Mediastinal Lymphadenopathy

**DOI:** 10.3390/arm94020019

**Published:** 2026-03-16

**Authors:** Paweł Zając, Monika Zając, Wojciech Kądziołka, Andrzej Sokołowski, Ewa Kaznowska

**Affiliations:** 1 Department of Thoracic Surgery, University of Rzeszów, 35-241 Rzeszów, Poland; wkchirklp@interia.pl; 2 Independent Researcher, 35-241 Rzeszów, Poland; zajac.monika.51@gmail.com; 3 Faculty of Management, Media and Technology, Andrzej Frycz Modrzewski Krakow University, 30-705 Kraków, Poland; asokolowski@uafm.edu.pl; 4 Department of Pathology, University of Rzeszów, 35-241 Rzeszów, Poland; ekaznowska3@gmail.com

**Keywords:** sarcoidosis, mediastinal lymphadenopathy, EBUS-TBNA, diagnostic factors, granulomatous disease, risk stratification

## Abstract

**Highlights:**

**What are the main findings?**

- In patients undergoing first-time EBUS-TBNA for mediastinal lymphadenopathy, younger age (≤55 years), female sex, absence of a pulmonary mass >10 mm, normal white blood cell count, and typical clinical features are associated with a higher likelihood of a definitive sarcoidosis diagnosis.
- A simple clinical scoring system based on routinely available pre-procedural data stratifies patients into low-, intermediate-, and high-probability diagnostic groups.

**What are the implications of the main findings?**

- The proposed scoring system may assist clinicians in estimating the likelihood of sarcoidosis before EBUS-TBNA and in discussing diagnostic expectations with patients.
- Identification of low- and high-probability groups may help guide the intensity of further diagnostic work-up and reduce unnecessary repeat invasive procedures.

**Abstract:**

Sarcoidosis is a multisystem granulomatous disease of unknown aetiology that frequently presents with mediastinal lymphadenopathy and often requires invasive diagnostic procedures. Endobronchial ultrasound-guided transbronchial needle aspiration (EBUS-TBNA) is widely used in this setting; however, a definitive diagnosis cannot always be established at first attempt. This study aimed to identify clinical, laboratory, and radiological factors associated with a definitive diagnosis of sarcoidosis in patients referred for EBUS-TBNA. A retrospective analysis was performed including patients undergoing first-time ever EBUS-TBNA for mediastinal lymphadenopathy over a 12-month period. Demographic data, clinical features suggestive of sarcoidosis, chest computed tomography findings, and white blood cell count, were analysed, and definitive diagnoses were established based on cytological results and available follow-up data. Younger age (≤55 years), female sex, the absence of a pulmonary mass >10 mm on imaging, normal white blood cell count, and the presence of clinical features typical of sarcoidosis were significantly associated with a definitive diagnosis of sarcoidosis. Based on these variables, two point-based diagnostic scoring models were developed, demonstrating clinically relevant discriminatory performance. Readily available pre-procedural clinical and radiological factors may assist in estimating the probability of sarcoidosis in patients undergoing EBUS-TBNA for mediastinal lymphadenopathy and may support risk stratification and clinical decision-making.

## 1. Introduction

Sarcoidosis is a multisystem granulomatous disease of unknown aetiology, characterised by the presence of non-caseating granulomas in affected organs. The most common manifestations include bilateral hilar lymphadenopathy and pulmonary parenchymal involvement. However, the disease may affect virtually any organ system and, in some cases, lead to significant deterioration in health status, permanent disability, or, in extreme cases, death. Despite extensive research, the aetiology of sarcoidosis remains unknown, and further investigation is required to optimise its detection, monitoring, and treatment.

At the Department of Thoracic Surgery of the Subcarpathian Centre for Pulmonary Diseases, EBUS-TBNA is frequently performed in patients presenting with mediastinal lymphadenopathy, and sarcoidosis is commonly established as the definitive diagnosis. Nevertheless, despite the relatively high sensitivity of this technique, repeat EBUS-TBNA procedures are sometimes required, and in certain cases a definitive diagnosis cannot be established even after multiple attempts. This may necessitate mediastinoscopy or surgical lung biopsy, both of which are associated with an increased risk of complications. Additionally, some patients, particularly those who are asymptomatic, may discontinue further diagnostic work-up due to frustration related to repeatedly non-diagnostic biopsy results, which in certain scenarios may result in disease progression due to the absence of appropriate treatment.

In cytological material obtained by EBUS-TBNA, pathologists search for multiple non-caseating epithelioid cell granulomas, which are characteristic of sarcoidosis. In some cases, these features are absent or only a single granuloma is identified, precluding a definitive diagnosis and prompting referral for repeat biopsies. The availability of additional markers or indicators that could support the diagnosis or exclusion of sarcoidosis without the need for further invasive procedures would therefore be highly beneficial. Even the identification of clinical and radiological factors associated with a higher likelihood of sarcoidosis could facilitate patient counselling and serve as an adjunctive tool in shared decision-making regarding further diagnostic steps.

## 2. Materials and Methods

A retrospective analysis was performed using data available in the hospital electronic medical records of patients referred for EBUS-TBNA at the Department of Thoracic Surgery. Over a 12-month period, from June 2021 to June 2022, a total of 274 patients underwent first-time ever EBUS-TBNA due to mediastinal lymphadenopathy. Only initial diagnostic procedures were included in the analysis.

Patients eligible for inclusion were consecutive adults (≥18 years) referred for planned diagnostic evaluation of mediastinal lymphadenopathy measuring ≥10 mm in short-axis diameter on chest computed tomography and undergoing first-time ever EBUS-TBNA during the study period. No additional study-specific exclusion criteria were applied beyond routine clinical qualification for elective diagnostic procedures. Patients presenting with clinical features suggestive of active infection were not admitted for planned hospitalisation and were therefore not included in the study cohort. All consecutive patients meeting referral criteria were included in the analysis, and patients without a confirmed disease diagnosis after available follow-up were retained as a separate outcome category.

For the purposes of the study, all data were anonymised and collected in a dedicated spreadsheet. The following variables were extracted from the medical records:

- Age.
- Presence of clinical features suggestive of sarcoidosis reported or observed in the weeks preceding hospital admission (including skin lesions, erythema nodosum, joint symptoms such as arthritis or arthralgia, and ocular involvement).
- Radiological findings on chest computed tomography (CT), categorised as:
  ○ Mediastinal lymphadenopathy associated with a single pulmonary mass >10 mm;
  ○ Mediastinal lymphadenopathy associated with multiple small pulmonary nodules ≤10 mm;
  ○ Isolated (“pure”) mediastinal lymphadenopathy without parenchymal lung abnormalities.
- White blood cell count (WBC), measured during hospitalisation.

Leukocytosis was defined as a WBC count >10 × 10^3^/µL, corresponding to the upper limit of normal used in the institutional laboratory.

Initial diagnoses were established according to pathology-driven clinical practice and based on cytological findings obtained during primary EBUS-TBNA.

A diagnosis of sarcoidosis required the presence of multiple non-necrotizing epithelioid granulomas, occasionally with multinucleated giant cells, identified in cytological or histopathological specimens and reported by the pathologist as consistent with sarcoidosis. Cases demonstrating only a single granuloma or non-specific granulomatous inflammation were considered insufficient for a definitive diagnosis and prompted further diagnostic evaluation.

Exclusion of infectious granulomatous diseases followed routine pathological assessment. When clinically indicated, additional microbiological or histochemical studies were performed, including Ziehl–Neelsen staining for Mycobacterium tuberculosis and special stains such as periodic acid–Schiff (PAS) or Grocott–Gomori methenamine silver (GMS) in cases of suspected fungal infection.

The diagnosis of malignancy required cytological or histopathological confirmation of malignant cells. Immunohistochemical studies were performed when necessary to establish tumour type, including markers such as TTF-1 and p40 for non-small cell lung carcinoma and synaptophysin, chromogranin A, or INSM1 in suspected neuroendocrine tumours.

When EBUS-TBNA findings were non-diagnostic or demonstrated benign changes (e.g., anthracotic lymph nodes), subsequent patient management was reconstructed from institutional medical records. Patients were routinely invited for repeat EBUS-TBNA according to local clinical practice; alternatively, when clinically indicated, surgical diagnostic procedures such as mediastinoscopy or lung parenchymal biopsy were performed. If any subsequent invasive procedure established a specific pathological diagnosis, this result was considered definitive. When repeat procedures remained non-diagnostic and no disease was subsequently identified, patients were classified as having no confirmed disease diagnosis.

In patients who did not undergo repeat EBUS-TBNA or an alternative invasive procedure, additional evaluation was reviewed to determine outcome classification. Radiological regression of mediastinal lymphadenopathy on follow-up imaging was interpreted as lack of evidence of active disease within the available follow-up period. Patients without further documented clinical evaluation or follow-up within the institutional system were classified as lost to follow-up and retained within the no confirmed disease diagnosis category.

Follow-up data were collected from electronic medical records covering a period from the initial EBUS-TBNA to the time of data extraction, with a maximum follow-up duration of approximately two years.

Statistical analyses were conducted to identify potential diagnostic factors associated with a definitive diagnosis of sarcoidosis.

Study groups were characterised using descriptive statistics, including arithmetic mean, median, and standard deviation. Differences between groups were assessed using the Student t-test for independent samples or non-parametric tests (Mann–Whitney U test and Kruskal–Wallis test) as appropriate.

Odds ratios (ORs) were estimated using logistic regression analysis. Initially, univariate logistic regression models were applied. Variables demonstrating significance at a level of *p* ≤ 0.10 in univariate analysis were subsequently entered into a multivariable logistic regression model. Model construction followed a backward stepwise regression approach, with sequential removal of variables exhibiting the highest *p*-values. The procedure was terminated when all remaining variables in the model reached statistical significance at *p* < 0.05.

Optimal cut-off values for continuous variables were determined based on ROC curve analysis, using the Youden index. The diagnostic performance of two point-based diagnostic models was evaluated using sensitivity, specificity, positive predictive value, and negative predictive value.

All statistical analyses and graphical presentations were performed using STATISTICA software, version 13.

## 3. Results

### 3.1. Study Population

The study population consisted of 274 patients, including 181 men and 93 women. Patient age ranged from 22 to 87 years, with a mean age of 60.0 years.

A single pulmonary mass was identified in 127 patients, multiple small pulmonary nodules in 46 patients, and isolated mediastinal lymphadenopathy without parenchymal lung abnormalities in 101 patients. White blood cell (WBC) counts ranged from 0.47 to 38.90 × 10^3^/µL, with a mean value of 8.43 × 10^3^/µL. According to institutional laboratory standards, the normal reference range for WBC was 4–10 × 10^3^/µL.

Clinical features potentially suggestive of sarcoidosis were reported by 31 patients: erythema nodosum in 19 patients, other skin lesions in 3 patients, joint symptoms in 16 patients, and anterior uveitis in 1 patient. Eight patients presented with two or more of the above symptoms simultaneously.

### 3.2. Definitive Diagnoses

Based on cytological findings from EBUS-TBNA and available follow-up data, the following definitive diagnoses were established: non-small cell lung cancer (*n* = 94), sarcoidosis (*n* = 87), small cell lung cancer (*n* = 23), lymphoproliferative disorders (*n* = 5), and tuberculosis (*n* = 2).

Overall, a definitive disease diagnosis was established in 211 patients, including 180 patients diagnosed based on the index EBUS-TBNA procedure. In 63 patients, no specific disease was identified; cytological findings were limited to normal, anthracotic, or reactive lymph nodes.

Among these 63 patients, 20 underwent subsequent invasive diagnostic procedures that remained non-diagnostic, 6 demonstrated radiological regression of lymphadenopathy, and 37 had no further follow-up data available in the electronic medical records. Based on these findings, all 63 patients were classified as patients without a confirmed disease diagnosis. A detailed list of definitive diagnoses is presented in Table 1.

### 3.3. Demographic Characteristics and White Blood Cell Count

Sex distribution, mean age, WBC levels, and the proportion of patients with leukocytosis were analysed across major diagnostic groups and are presented in Table 2.

In most diagnostic categories, the male-to-female ratio approximated 2:1. Minor deviations from this pattern were observed in the sarcoidosis group (57% men, 43% women) and a marked deviation in the tuberculosis group, which consisted exclusively of women.

Mean patient age differed between diagnostic groups. The lowest mean age was observed in patients with sarcoidosis (46.4 years), followed by patients with malignancy (67.92 years), while the highest mean age was noted in patients with tuberculosis (83.5 years).

Mean WBC levels ranged from 6.84 × 10^3^/µL in the sarcoidosis group to 9.95 × 10^3^/µL in patients with non-small cell lung cancer and 13.15 × 10^3^/µL in patients with lymphoproliferative disorders. The proportion of patients with leukocytosis also differed between groups.

Of all 274 patients, 58 (21.2%) presented with leukocytosis at the time of qualification for EBUS-TBNA. Only 9.2% of patients in the sarcoidosis group had leukocytosis, compared with an average of 30.3% in the oncological group, with the highest proportion observed in non-small cell lung cancer. The presence of leukocytosis influenced the distribution of definitive diagnoses.

**Figure 1:**
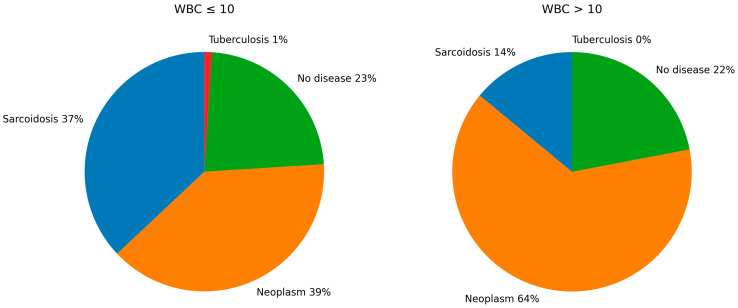
Distribution of diagnoses by white blood cell count at admission.

Among patients with a normal WBC count, the probability of sarcoidosis, malignancy, tuberculosis, and no confirmed disease diagnosis was 36.6%, 39.4%, 0.9%, and 23.2%, respectively. In contrast, among patients presenting with leukocytosis, the corresponding probabilities were 13.8%, 63.8%, 0.0%, and 22.4%. These distributions are illustrated in Figure 1.

### 3.4. Radiological Findings

Among the 101 patients presenting with isolated mediastinal lymphadenopathy, the majority were diagnosed with sarcoidosis (*n* = 56). No specific disease was confirmed in 35 patients, malignancy was diagnosed in 9 patients, and tuberculosis in 1 patient.

In patients with mediastinal lymphadenopathy accompanied by a single pulmonary mass >10 mm, malignant disease predominated. Of the 127 patients in this category, 101 were diagnosed with neoplastic disease. The remaining 26 patients included those without a confirmed disease diagnosis (*n* = 20), patients with sarcoidosis (*n* = 5), and one patient with tuberculosis.

Among the 46 patients with mediastinal lymphadenopathy associated with multiple small pulmonary nodules, sarcoidosis was diagnosed in 26 patients, malignancy in 12 patients, and no specific disease in 8 patients. These data are summarised in Table 3.

### 3.5. Clinical Features Typical of Sarcoidosis

Among the 31 patients who reported clinical features considered typical of sarcoidosis, 29 ultimately received a definitive diagnosis of sarcoidosis. This included all patients presenting with erythema nodosum, skin lesions, or ocular involvement, as well as the majority of patients with joint symptoms (14 of 16). One patient with arthritis was diagnosed with metastatic cancer, and in one patient no specific disease was identified.

### 3.6. Diagnostic Factors Associated with Sarcoidosis

#### 3.6.1. Sex

Sarcoidosis was diagnosed in 39.78% of women referred for EBUS-TBNA, compared with 27.62% of men. Female sex was therefore associated with a definitive diagnosis of sarcoidosis (*p* = 0.0406).

#### 3.6.2. Age

**Figure 2:**
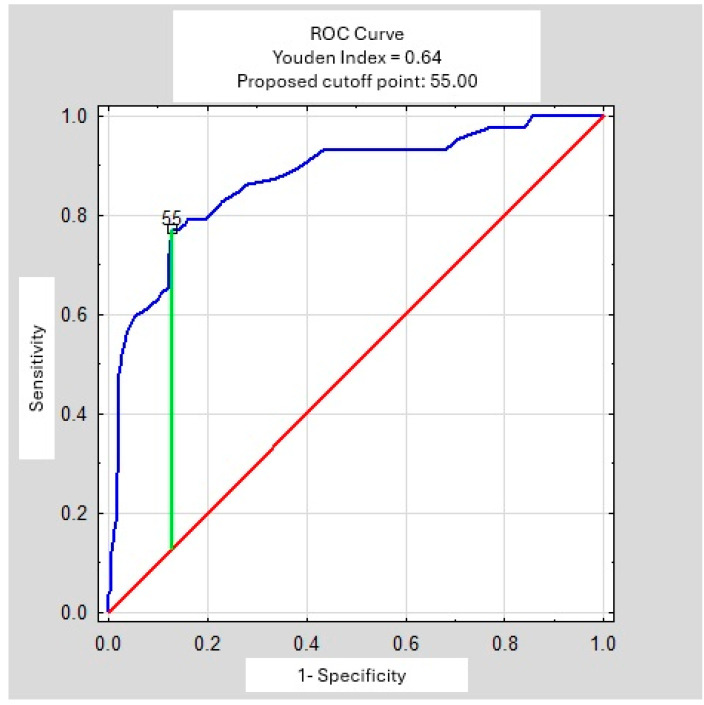
ROC Curve—age (years).

In both the Student *t*-test and the Mann–Whitney U test, mean age differed between patients with sarcoidosis and the remaining study population, with an approximate difference of 20 years (*p* < 0.001) (Table 4). ROC curve analysis suggested an optimal age cut-off of 55 years (Figure 2).

Among patients aged ≤55 years (*n* = 91), 73.63% (*n* = 67) were diagnosed with sarcoidosis (*p* < 0.001).

#### 3.6.3. Leukocytosis

**Figure 3:**
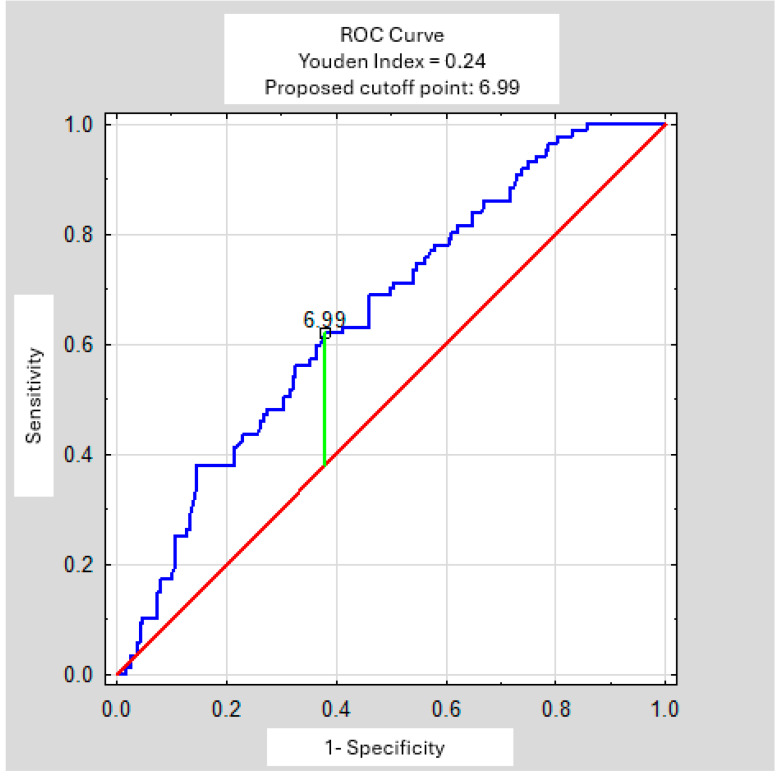
ROC Curve—White Blood Count (×10^3^/µL).

In both the Student *t*-test and the Mann–Whitney *U* test, the mean WBC level in patients with sarcoidosis differed significantly from that in the remaining study participants (*p* = 0.0000) (Table 4). The ROC curve indicates a WBC cutoff point at approximately 7 × 10^3^/µL; however, the curve is relatively flat, which allows for some flexibility in threshold selection (Figure 3). Therefore, for the purposes of the proposed scale, we suggest adopting the standard upper limit used in the laboratory, which in our case is 10 × 10^3^/µL.

Leukocytosis identified at the time of initial evaluation is a strong negative factor for the diagnosis of sarcoidosis. In the studied population, the presence of leukocytosis was associated with an approximately threefold reduction in the probability of establishing this diagnosis (from 36.57% to 13.79%).

#### 3.6.4. Clinical Features Typical of Sarcoidosis

The presence of clinical features typical of sarcoidosis was strongly associated with a definitive diagnosis. Of the 31 patients reporting such symptoms, 29 were ultimately diagnosed with sarcoidosis.

#### 3.6.5. Radiological Findings

Sarcoidosis predominated among patients presenting with isolated mediastinal lymphadenopathy and among those with lymphadenopathy accompanied by multiple small pulmonary nodules. In total, 147 patients presented with these radiological patterns, and non-caseating epithelioid cell granulomas were identified in 82 of them.

The presence of a pulmonary mass >10 mm on imaging was negatively associated with a diagnosis of sarcoidosis (Table 5).

### 3.7. Statistical Modelling and Scoring Systems

Table 6 and Table 7 present the analysed variables along with their odds ratios and corresponding *p*-values. The strongest diagnostic factors for sarcoidosis were age ≤55 years (OR = 22.75), absence of a pulmonary mass >10 mm (OR = 30.78), and presence of clinical features typical of sarcoidosis (OR = 46.25). These variables were assigned 2 points each in the proposed scoring system.

Weaker, yet statistically significant factors—female sex (OR = 1.73) and WBC < 10 × 10^3^/µL (OR = 3.60)—were assigned 1 point each. The total score ranged from 0 to 8 points.

Application of this scoring system to the study population yielded the distribution presented in Table 8.

**Figure 4:**
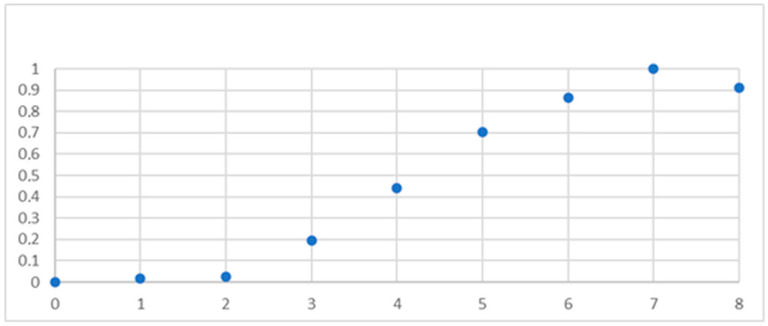
Percentage of sarcoidosis diagnoses according to the number of points.

Except for the extreme values, the proportion of patients diagnosed with sarcoidosis increased progressively with rising scores, approximating a linear relationship (Figure 4).

Two point-based diagnostic models were subsequently proposed, and their diagnostic performance at selected cut-off values is summarised in Table 9.

Model I divided patients into low-risk (<5 points) and high-risk (≥5 points) groups. Among patients scoring <5 points (*n* = 193), only 10.88% (*n* = 21) were diagnosed with sarcoidosis. In contrast, among patients scoring ≥5 points (*n* = 81), sarcoidosis was diagnosed in 81.48% (*n* = 66).

Model II stratified patients into three risk categories: low risk (0–2 points), intermediate risk (3–5 points), and high risk (6–8 points). The corresponding proportions of sarcoidosis diagnoses were 1.57%, 43.69%, and 90.91%, respectively.

The advantages and limitations of both models are addressed in detail in Section 4.

## 4. Discussion

In the analysed cohort, men predominated, with a male-to-female ratio of 57:43%. This distribution falls within the range reported in previous studies, although the literature indicates substantial variability in sex distribution depending on the population studied. For example, in the study by Filarecka et al., men accounted for 66% and women for 34% of the cohort [1], whereas in the study by Ribeiro et al., women constituted 56.4% and men 43.6% of patients [2].

It is well established that sarcoidosis occurs more frequently in women, which has been attributed to complex hormonal, immunological, and genetic mechanisms. The incidence in women peaks between the fifth and sixth decades of life, corresponding to the perimenopausal period, suggesting a significant role of oestrogens in disease pathogenesis [3]. Population-based studies have demonstrated that menopausal hormone therapy is associated with an increased risk of sarcoidosis [3]. Furthermore, women exhibit a stronger Th1- and Th17-mediated cellular immune response, which may predispose to granuloma formation [4]. Oestrogens modulate immune system activity in a dose-dependent manner and may exert both pro-inflammatory and immunosuppressive effects [5]. In addition, immune-related genes located on the X chromosome and sex-specific epigenetic differences may enhance immune reactivity in women and increase susceptibility to autoimmune and granulomatous diseases, including sarcoidosis [6].

In the present study, the mean age at diagnosis of sarcoidosis was 46.35 years, which is consistent with reports from other cohorts. In a Polish multicentre study, the mean age was 42 years [1]. Similar values were reported in Portuguese studies, with Neves et al. reporting a median age of 45 years [7] and Ribeiro et al. a mean age of 46.7 years [2]. Likewise, Wong et al., analysing patients diagnosed using EBUS, reported a mean age of 45 years [8]. These findings indicate a relatively consistent age range for sarcoidosis diagnosis, most commonly between the fourth and fifth decades of life.

The most frequent radiological manifestation of thoracic sarcoidosis is bilateral hilar and mediastinal lymphadenopathy; however, multiple perilymphatic pulmonary nodules are also commonly observed on computed tomography [9]. These lesions typically present as micronodules with a diameter below 4 mm, although they may coalesce into larger conglomerates [10]. In the study by Koo et al., which analysed 226 patients with larger nodules (macronodules), the mean nodule size was 6.3 mm, with the smallest measuring 5.1 mm and the largest 13 mm [11]. Conversely, Kurogouchi et al. reported that nodules exceeding 10 mm were observed in only 2–4% of sarcoidosis cases [12], suggesting that in the vast majority of patients, pulmonary nodules remain below 1 cm. Therefore, adopting a 10 mm cut-off for defining a pulmonary mass appears clinically justified and consistent with existing evidence.

Leukopenia and lymphopenia are frequently observed in sarcoidosis and are thought to reflect peripheral consumption or redistribution of lymphocytes to affected tissues. This contrasts with other granulomatous and infectious diseases, in which leukocytosis is more commonly observed [13]. Additional haematological abnormalities, such as anaemia or thrombocytopenia, may also occur and are usually associated with hypersplenism or bone marrow involvement by granulomas [14].

In the literature, lymphopenia has received considerably more attention than leukopenia and has been the focus of numerous recent studies. Phenotypic analyses have shown that reduced peripheral blood lymphocyte counts correlate with increased inflammatory activity on ^18^F-FDG PET/CT, suggesting that lymphopenia may serve as a marker of sarcoidosis activity [15]. Similar observations were reported in a large Swedish cohort, in which lymphopenia was more frequent in patients with a poorer prognosis. No significant association was found between lymphocyte counts in bronchoalveolar lavage fluid and extrapulmonary involvement; however, lymphopenia was associated with carriage of the HLA-DRB1*07 allele, a higher likelihood of chronic disease, and the need for immunosuppressive therapy [16].

Lymphocyte counts were not analysed separately, as differential blood counts were not consistently available for all patients at the time of qualification for EBUS-TBNA.

Patients referred for EBUS-TBNA in the present cohort reported a wide range of non-specific symptoms, including cough, fatigue, low-grade fever, dyspnoea, and chest pain. In the context of mediastinal lymphadenopathy, these symptoms are not specific to a particular aetiology and were therefore not analysed in detail. Instead, emphasis was placed on symptoms considered more characteristic of sarcoidosis than of malignant disease.

It is estimated that approximately one quarter of patients with sarcoidosis develop cutaneous manifestations during the course of the disease. Specific skin lesions associated with non-caseating epithelioid cell granulomas include lupus pernio, red-brown plaques, maculopapular eruptions, subcutaneous nodules, and scar sarcoidosis. Non-specific skin manifestations, most notably erythema nodosum, are also observed and constitute a component of Löfgren’s syndrome [17].

Given its particular diagnostic relevance, erythema nodosum was analysed separately from other skin manifestations. Clinical studies estimate that erythema nodosum occurs in approximately 10–20% of patients with sarcoidosis, and its presence significantly increases the likelihood of diagnosis, especially when accompanied by systemic symptoms such as fever or joint pain and by lymphadenopathy [13]. In the present cohort, erythema nodosum was reported by 19 patients and other skin lesions by 3 patients, corresponding to 21.8% and 3.4%, respectively, of all patients diagnosed with sarcoidosis (***n*** = 87). These findings are consistent with previously published data.

Musculoskeletal manifestations of sarcoidosis include both arthralgia and inflammatory arthritis, with reported prevalence varying widely across studies and populations. Day and Hamann reported musculoskeletal involvement in 10–40% of patients [18]. Similarly, Yeung et al., in a systematic review and meta-analysis, found that approximately 12% of patients had joint involvement, with cumulative prevalences of 19% for arthritis and 32% for arthralgia [19]. In the present study, 14 patients reported joint symptoms, accounting for 16% of all patients diagnosed with sarcoidosis, which aligns with the ranges reported in the literature.

Ocular involvement in sarcoidosis has been reported in 25–60% of patients with systemic disease, depending on the population studied and the extent of ophthalmological evaluation. Uveitis is the most common manifestation, occurring in 30–70% of patients with ocular involvement [20]. More recent analyses suggest a prevalence of 20–30%, reflecting differences in diagnostic criteria and population characteristics [21]. In the present cohort, ocular symptoms were documented in only one patient, which may indicate underreporting or insufficiently detailed ophthalmological history-taking at the time of qualification for EBUS-TBNA.

Certain potentially relevant confounders, such as smoking status or ethnicity, were not included in the analysis, as these variables were not consistently available in the retrospective dataset. Moreover, the study population was relatively ethnically homogeneous, which likely limits the impact of ethnicity as a confounding factor. Nevertheless, the absence of these variables represents a limitation of the present analysis and should be considered when interpreting the results.

A proportion of patients in the cohort did not have a confirmed disease diagnosis and some were lost to follow-up within the institutional system. Although these cases were retained to preserve the consecutive real-world nature of the cohort, it cannot be excluded that a small number of these patients may have had an undiagnosed underlying condition. This represents a potential source of outcome misclassification and should be considered when interpreting the results.

The two proposed scoring models were designed to address different clinical and research objectives.

Model I divides the study population into two groups. Among patients who scored 5 points or more (*n* = 81), the vast majority received a definitive diagnosis of sarcoidosis. Only 15 patients were diagnosed with alternative conditions, including metastatic adenocarcinoma (*n* = 2), Hodgkin lymphoma (*n* = 1), or were classified as having no confirmed disease diagnosis or lacking follow-up data (*n* = 12). Importantly, among patients selected using this threshold, only three individuals received a definitive diagnosis other than sarcoidosis.

It should be noted that among patients who did not attend further recommended follow-up visits, a proportion might ultimately have been diagnosed with sarcoidosis. If this were the case, the proportion of sarcoidosis diagnoses in this high-risk group could potentially exceed 90%. Consequently, the use of a cut-off value of 5 points may serve as an effective pre-diagnostic screening tool for research planning, particularly for the selection of study populations and recruitment of patients for further clinical or translational studies.

Clinical research often requires substantial investments of time and financial resources. Therefore, the ability to preselect, using simple and readily available clinical data, a group of patients with a very high probability of sarcoidosis may significantly reduce research costs. Such an approach may facilitate studies employing expensive or limited-access reagents by restricting their use to a preselected cohort that nonetheless remains sufficiently diverse in terms of demographic characteristics.

Model II appears particularly well suited for use in routine clinical practice. Stratifying patients into three risk categories according to predefined score thresholds allows for an initial estimation of the probability of sarcoidosis while simultaneously reflecting the distribution of alternative diagnoses.

In the low-risk group (0–2 points), the vast majority of patients were diagnosed with metastatic malignant disease (78.74%), while sarcoidosis accounted for less than 2% of diagnoses. In contrast, in the high-risk group (6–8 points), this proportion was completely reversed: 90.91% of patients were diagnosed with sarcoidosis, and no cases of malignant disease were identified.

Such a strong association between extreme score categories and definitive diagnoses may substantially support optimal clinical decision-making. It may also facilitate communication with patients prior to planned EBUS-TBNA, reduce uncertainty during the diagnostic process, and shorten waiting times for further consultations or invasive diagnostic procedures in patients for whom delays in treatment initiation may have clinically significant consequences.

The intermediate-risk group (3–5 points) was the most heterogeneous, with 43.69% of patients diagnosed with sarcoidosis and 21.36% diagnosed with malignant disease based on lymph node pathology. Although this group is less uniform, the near-linear increase in the proportion of sarcoidosis diagnoses with rising scores suggests that even within this range, the scoring system may provide meaningful guidance for clinicians.

## 5. Conclusions

Younger age (≤55 years), female sex, absence of a pulmonary mass >10 mm on imaging, normal white blood cell count, and the presence of clinical features typical of sarcoidosis constitute significant diagnostic factors associated with a high likelihood of sarcoidosis in patients undergoing EBUS-TBNA for mediastinal lymphadenopathy. Identification of these factors may enable more accurate pre-procedural risk stratification, improve communication with patients, and reduce the need for repeated invasive diagnostic procedures. The proposed scoring system requires further validation in prospective studies.

## Figures and Tables

**Table 1:** Final diagnoses established in the study population.

Diagnosis	Quantity
**Entire study population**	274
**No confirmed disease diagnosis**	63
**Any confirmed disease**	211
Sarcoidosis	87
Tuberculosis	2
Malignancy (total)	122
Non-small cell lung cancer (total)	94
NSCLC not otherwise specified	29
Adenocarcinoma	36
Squamous cell carcinoma	27
Large cell carcinoma	2
Small cell lung cancer	23
Lymphoproliferative disorders (total)	5
Hodgkin lymphoma	3
Other lymphoproliferative disorders	2

**Table 2:** Demographic characteristics and white blood cell count according to final diagnosis.

Final Diagnosis	Male (%)	Female (%)	Mean Age (Years)	Mean WBC ^1^ (×10^3^/µL)	Leukocytosis ^2^ (%)
**Sarcoidosis**	57.0	43.0	46.4	6.8	9.2
**Non-small cell lung cancer**	68.1	31.9	67.9	10.0	33.0
**Small cell lung cancer**	69.6	30.4	66.4	10.2	30.4
**Lymphoproliferative disorders**	60.0	40.0	71.2	13.2	40.0
**Tuberculosis**	0.0	100.0	83.5	8.6	0.0
**No confirmed disease diagnosis**	61.9	38.1	62.1	7.9	14.3

^1^ WBC—white blood cell count. ^2^ Leukocytosis defined as WBC > 10 × 10^3^/µL.

**Table 3:** Radiological patterns on chest computed tomography and final diagnoses.

Radiological Pattern	Sarcoidosis	Malignancy	Tuberculosis	No Disease ^1^	Total
**Isolated lymphadenopathy**	56	9	1	35	101
**Lymphadenopathy with nodules ^2^**	26	12	0	8	46
**Lymphadenopathy with tumour ^3^**	5	101	1	20	127
**Total**	87	122	2	63	274

^1^ No confirmed disease diagnosis. ^2^ Mediastinal lymphadenopathy with multiple pulmonary nodules ≤10 mm. ^3^ Mediastinal lymphadenopathy with a single pulmonary mass >10 mm.

**Table 4:** Comparison of age and white blood cell count between patients with sarcoidosis and other diagnoses.

	Quantity		Mean		Standard Deviation		*p*-Value	
	Sarcoidosis	No Sarc.	Sarcoidosis	No Sarc.	Sarcoidosis	No Sarc.	Student *t*-Test	Mann–Whitney U Test
**Age (years)**	87	187	46.4	66.4	13.3	10.4	0.0000	0.0000
**WBC (×10^3^/µL)**	87	187	6.84	9.18	2.09	5.20	0.0000	0.0000

**Table 5:** Association between the presence of a pulmonary mass >10 mm on chest computed tomography and the diagnosis of sarcoidosis (*p* = 0.0000).

	Sarcoidosis	No Sarcoidosis
**No tumour**	82 (55.8%)	65 (44.2%)
**Tumour ^1^ present**	5 (3.9%)	122 (96.1%)

^1^ Tumour—defined as a solid lesion >10 mm.

**Table 6:** Logistic regression—univariate analysis.

Variable	Category	*n*	OR	95% CI	*p* Value
**Sex**	Male	181	1.00	Reference	–
	Female	93	1.73	1.02–2.94	0.0416
**Age**	≤55 years	91	22.75	11.74–44.07	0.0000
	>55 years	183	1.00	Reference	–
**Single tumour > 10 mm**	Yes	127	1.00	Reference	–
	No	147	30.78	11.83–80.07	0.0000
**Nodules ≤ 10 mm**	Yes	46	3.56	1.85–6.85	0.0001
	No	228	1.00	Reference	–
**Isolated lymphadenopathy**	Yes	101	5.70	3.27–9.93	0.0000
	No	173	1.00	Reference	–
**Clinical features**	Yes	31	46.25	10.63–201.08	0.0000
	No	243	1.00	Reference	–
**WBC < 7 × 10^3^/µL**	Yes	125	2.67	1.58–4.53	0.0002
	No	149	1.00	Reference	–
**WBC < 10 × 10^3^/µL**	Yes	216	3.60	1.62–8.02	0.0016
	No	58	1.00	Reference	–

CI—confidence interval; OR—odds ratio; WBC—white blood cell count.

**Table 7:** Logistic regression—multivariable analysis.

Variable	Category	*n*	Odds Ratio (OR)	95% CI	*p* Value
**Age**	≤55 years	91	10.95	2.06–23.70	<0.001
	>55 years	183	1.00	Reference	–
**Single tumour > 10 mm**	Yes	127	1.00	Reference	–
	No	147	15.45	5.36–44.57	<0.001
**Clinical features**	Yes	31	10.86	2.23–52.98	0.003
	No	243	1.00	Reference	–

CI—confidence interval; OR—odds ratio.

**Table 8:** Distribution of sarcoidosis diagnoses according to total score and proposed point-based models.

Total Score	Number of Patients	Sarcoidosis Diagnoses (*n*)	Sarcoidosis Diagnoses (%)	Model I	Model II
**0**	25	0	0.00	Low risk	Low risk
**1**	62	1	1.61	Low risk	Low risk
**2**	40	1	2.50	Low risk	Low risk
**3**	41	8	19.51	Low risk	Intermediate risk
**4**	25	11	44.00	Low risk	Intermediate risk
**5**	37	26	70.27	High risk	Intermediate risk
**6**	22	19	86.36	High risk	High risk
**7**	11	11	100.00	High risk	High risk
**8**	11	10	90.91	High risk	High risk

Model I: low risk <5 points; high risk ≥5 points. Model II: low risk 0–2 points; intermediate risk 3–5 points; high risk 6–8 points.

**Table 9:** Diagnostic performance of the point-based scoring system for sarcoidosis at selected cut-off values.

Cut-Off Value	Sensitivity	Specificity	PPV	NPV
**≥3 points**	0.977	0.668	0.578	0.984
**≥4 points**	0.885	0.845	0.726	0.940
**≥5 points**	0.759	0.920	0.815	0.891
**≥6 points**	0.460	0.979	0.909	0.796

PPV—Positive predictive value. NPV—Negative predictive value.

## Data Availability

The data presented in this study are available on request from the corresponding author due to privacy reasons.

## Abbreviations

The following abbreviations are used in this manuscript:

CI	Confidence interval
CT	Computed tomography
EBUS	Endobronchial ultrasound
EBUS-TBNA	Endobronchial ultrasound-guided transbronchial needle aspiration
^18^F-FDG PET/CT	Fluorodeoxyglucose positron emission tomography/computed tomography
HLA	Human leukocyte antigen
NSCLC	Non-small cell lung cancer
NPV	Negative predictive value
OR	Odds ratio
PET/CT	Positron emission tomography/computed tomography
PPV	Positive predictive value
ROC	Receiver operating characteristic
WBC	White blood cell count
